# Attributional styles and other cognitive biases in patients with delusional disorder: A systematic review

**DOI:** 10.1192/j.eurpsy.2023.580

**Published:** 2023-07-19

**Authors:** A. Guàrdia, A. González-Rodríguez, M. V. Seeman, X. Martinez-Bio, M. Natividad, J. A. Monreal

**Affiliations:** ^1^Psychiatry, Hospital Universitari Mutua de Terrassa, Terrassa, Spain; ^2^Psychiatry, Hospital Toronto, Toronto, Canada

## Abstract

**Introduction:**

The accurate examination of attributional patterns and cognitive biases in delusional patients is relevant to explain the externalizing tendency in paranoid schizophrenia patients. In subjects with delusional disorder (DD), attributional styles and other cognitive bias have been poorly investigated.

**Objectives:**

Our main goal was to review the tendency to use external-internal attributions for negative events and the presence/absence of other cognitive biases in patients suffering from DD.

**Methods:**

A systematic review was conducted in PubMed and ClinicalTrials.gov databases/registers up to September 2022 according to the PRISMA Guidelines. The following key-words were searched in the title and abstracts: (attributions OR attributional OR “cognitive” OR “cognition” OR “social cognition”) AND (“delusional disorder”). Additionally, references of included studies were manually examined to identify further studies.

**Results:**

A total of 144 records were identified (Pubmed, n=125; ClinicalTrials.gov, n=16; other sources, n=13), five studies met our inclusion criteria, reporting attributional styles (n=5) and other cognitive biases (n=2) in DD. (A)Attributional style in DD. Mainly excessive external attributions implying the ascribing of negative experiences to another person’s behavior or action. Other authors describe attributions of negative events to internal causes (n=2). (B)Cognitive biases: Jumping to conclusions bias or judgments made on inadequate evidence have been described in DD (n=2).

**Conclusions:**

Findings in attributional patterns in DD are mixed. Several authors report external and stable attributions in DD, whereas others described internal attributes for negative events, suggesting that depressive vs. “pure” paranoid core dimensions may appear in DD.

**Disclosure of Interest:**

None Declared

